# Immunomodulatory potential of mesenchymal stromal cell-derived extracellular vesicles in chondrocyte inflammation

**DOI:** 10.3389/fimmu.2023.1198198

**Published:** 2023-07-26

**Authors:** Robert Ossendorff, Sibylle Grad, Tobias Tertel, Dieter C. Wirtz, Bernd Giebel, Verena Börger, Frank A. Schildberg

**Affiliations:** ^1^ Department of Orthopedics and Trauma Surgery, University Hospital Bonn, Bonn, Germany; ^2^ AO Research Institute Davos, Davos, Switzerland; ^3^ Institute for Transfusion Medicine, University Hospital Essen, Essen, Germany

**Keywords:** extracellular vesicles, osteoarthritis, chondrocytes, mesenchymal stromal cells, tumor necrosis factor alpha, musculoskeletal immunology

## Abstract

**Introduction:**

Osteoarthritis (OA) affects a large percentage of the population worldwide. Current surgical and nonsurgical concepts for treating OA only result in symptom-modifying effects. However, there is no disease-modifying therapy available. Extracellular vesicles released by mesenchymal stem/stromal cells (MSC-EV) are promising agents to positively influence joint homeostasis in the osteoarthritic surroundings. This pilot study aimed to investigate the effect of characterized MSC-EVs on chondrogenesis in a 3D chondrocyte inflammation model with the pro-inflammatory cytokine TNFα.

**Methods:**

Bovine articular chondrocytes were expanded and transferred into pellet culture at passage 3. TNFα, human MSC-EV preparations (MSC-EV batches 41.5-EV_i1_ and 84-EV_i_), EVs from human platelet lysate (hPL_4_-EV), or the combination of TNFα and EVs were supplemented. To assess the effect of MSC-EVs in the chondrocyte inflammation model after 14 days, DNA, glycosaminoglycan (GAG), total collagen, IL-6, and NO release were quantified, and gene expression of anabolic (COL-II, aggrecan, COMP, and PRG-4), catabolic (MMP-3, MMP-13, ADAMTS-4 and ADAMTS-5), dedifferentiation (COL-I), hypertrophy (COL-X, VEGF), and inflammatory (IL-8) markers were analyzed; histological evaluation was performed using safranin O/Fast Green staining and immunohistochemistry of COL I and II. For statistical evaluation, nonparametric tests were chosen with a significance level of p < 0.05.

**Results:**

TNFα supplementation resulted in catabolic stimulation with increased levels of NO and IL-6, upregulation of catabolic gene expression, and downregulation of anabolic markers. These findings were supported by a decrease in matrix differentiation (COL-II). Supplementation of EVs resulted in an upregulation of the chondrogenic marker PRG-4. All MSC-EV preparations significantly increased GAG retention per pellet. In contrast, catabolic markers and IL-8 expression were upregulated by 41.5-EV_i1_. Regarding protein levels, IL-6 and NO release were increased by 41.5-EV_i1_. Histologic and immunohistochemical evaluations indicated a higher differentiation potential of chondrocytes treated with 84-EV_i_.

**Discussion:**

MSC-EVs can positively influence chondrocyte matrix production in pro-inflammatory surroundings, but can also stimulate inflammation. In this study MSC-EV 41.5-EV_i1_ supplementation increased chondrocyte inflammation, whereas MSC-84-EV_i_ supplementation resulted a higher chondrogenic potential of chondrocytes in 3D pellet culture. In summary, the selected MSC-EVs exhibited promising chondrogenic effects indicating their significant potential for the treatment of OA; however, the functional heterogeneity in MSC-EV preparations has to be solved.

## 1 Introduction

Osteoarthritis (OA) is a degenerative joint disease affecting a large percentage of the population worldwide (1). It is characterized by synovial inflammation, cartilage degradation, and subchondral bone affection with typical symptoms of knee swelling, pain, and loss of function. A central hallmark of disease progression is the elevated concentration of pro-inflammatory cytokines, such as tumor necrosis factor alpha (TNFα), interleukin (IL)-1ß, and IL-6 (2). There is an urgent need for therapeutics that counteract the inflammatory signals and halt or reverse cartilage degradation. However, only symptom-modifying intraarticular injected drugs for the treatment of osteoarthritis are available, including hyaluronic acid and glucocorticoids (3). Historically, mesenchymal stromal cells (MSCs) were considered as ideal candidates for cartilage regenerative therapy in OA based on the described chondrogenic differentiation potential *in vitro* (4). According to the ISCT definition MSCs are plastic-adherent cells expressing the surface markers CD105, CD73, CD90, CD146, CD29 and absence of CD45, CD34, CD14, CD19, HLA-DR, poor expression of MHC I molecules, and with the ability to differentiate into mesodermal and non-mesodermal cells like chondrocytes, osteocytes, cardiomyocytes, adipocytes, and neural cells *in vitro* (5, 6). These *in vitro* findings could not be detected *in vivo*. Murphy et al. reported in a caprine model of OA by anterior cruciate ligament and medial meniscus resection that after intraarticular injection of bone marrow derived MSCs a large percentage (97%) disappeared after a few days and most of the remaining cells were located in the synovium (7). Nevertheless, they could detect induction of knee joint regeneration including meniscus regeneration and reduction of cartilage degeneration, osteophytic remodeling, and subchondral sclerosis compared to the non-treated controls. These regenerative effects can be explained by the paracrine effect of MSCs (8). MSCs induce recruitment of endogenous MSCs by direct cross-talk, modulation of immune system cells and the MSCs secretome (9). The MSC secretome includes multiple signaling molecules with an immunomodulatory, anti-catabolic, anti-apototic effect resulting in a chondrogenic stimulus with endogenous cartilage repair and differentiation (10). Current studies have demonstrated that most of the therapeutic potential of MSCs is driven by their secretome (9). Extracellular vesicles (EVs) are a central component of the MSCs secretome. They are a heterogeneous group of exosomes, microvesicles, and apoptotic bodies with sizes between 30 and 5000 nm, and encapsulated by a lipid bilayer (11). EVs play a central role in immunoregulation through cell-cell interaction with specific EV cargo, such as RNA, protein and lipids. Initial *in vitro* and preclinical *in vivo* studies of MSC-EVs showed a high potential to positively modulate joint homeostasis (12). Tofino-Vian et al. (13) reported in an *in vitro* culture of human OA chondrocytes stimulated by the proinflammatory cytokine IL-1ß (10ng/ml) increased inflammation and elevated activity of catabolic factors. The supplementation of adipose derived MSC-EVs induced a decrease of inflammatory mediators and catabolic enzymes and increased expression of the anti-inflammatory cytokine IL-10 in chondrocyte culture. Similar *in vitro* studies denote the potential of MSC-EVs to modulate the chondrogenic differentiation of articular chondrocytes (14, 15). Consequently, mesenchymal stem/stromal cell-derived extracellular vesicles (MSC-EV) are being discussed as a novel therapeutic approach to OA (16). Due to a lack of standardization in MSC culture, EV production, and EV characterization, it is difficult compare these previous studies and to enable translation into clinical applications (17). All these factors can influence the functional phenotype of MSC-EVs and their therapeutical efficacy.

This pilot study aimed to investigate the effect of repeated supplementation of characterized MSC-EV preparations on chondrogenesis in a 3D chondrocyte pellet culture model over 14 days. In addition, we aimed to analyze the therapeutic potential of MSC-EVs in a standardized inflammation model using the pro-inflammatory cytokine TNFα. A broad gene expression and protein analysis of anabolic, catabolic and inflammatory factors was performed to analyze the chondrogenic potential of the stimulated chondrocytes of 3D pellet constructs.

## 2 Materials and methods

### 2.1 MSC-EV isolation and characterization

MSCs initially raised from bone marrow samples of two healthy human donors after informed consent (18); the study was approved by the local ethical commission with approval number 12-5295-BO as described before (19). The stocks of MSC 41.5 and MSC 84 were expanded at 37°C, 5% CO_2_ using Dulbecco´s modified Eagle’s medium (DMEM) low glucose basal medium (PAN Biotech, Aidenbach, Germany), 10% human platelet lysate (hPL; in house production; batch hPL_4_), 100 U/mL penicillin-streptomycin-glutamine (Thermo Fisher Scientific, Waltham, USA), and 5 IU/mL Heparin (Ratiopharm, Ulm, Germany) (20). Medium change was started at 50% confluency and performed every 48 h until cells reached a density of ~80%, following passaging. EVs from conditioned-media of MSCs (MSC-EVs) and non-conditioned media (hPL-EVs) were isolated using an optimized polyethylene glycol 6000 precipitation protocol and ultracentrifugation as described previously (20, 21). Subsequently, CM of 4 × 10^7^ MSCs were resuspended in 1 mL 10 mM HEPES/0.9% NaCl buffer (Thermo Fisher Scientific) and stored at –80°C. MSC-EV preparations were characterized according to the minimal information for studies of extracellular vesicles 2018 (MISEV 2018) (22), including nanoparticle tracking analysis on a ZetaView (Particle Metrix GmbH, Meerbusch, Germany) for measurement of size and concentration as well as imaging flow cytometry to analyze the EV markers CD9, CD59, CD63, and CD81 as previously described (23). Imaging flow cytometry is described in more detail in the **supplementary materials** (**Supplementary Methods**). A multi-donor mixed lymphocyte reaction assay (mdMLR) was performed to characterize the MSC-EV preparations for *in vitro* T-cell immunomodulatory activity as described previously (18). Preparations were labeled according to their immunomodulatory potential and batch number (EV_a_, active; EV_i_, inactive).

### 2.2 Isolation and culture of bovine chondrocytes

Articular chondrocytes were harvested from the fetlock joints of 4–6 months old calves, which were euthanized on the same day by a local butcher. The calves were euthanized for food production and the fetlock joints were wasted without the need for ethical approval. The cartilage was cut into pieces of < 25 mm² in size, predigested with pronase (0.1%; Merck, Darmstadt, Germany) for 105min., and digested in collagenase 2 (Worthington, Lakewood, USA) for 14 h. The chondrocytes were seeded at a density of 16.7 × 10^3^ cells/cm^2^ in DMEM (high glucose) (Thermo Fisher Scientific) with 10% FBS (Bio & Sell, Feucht, Germany). The cells were passaged at 90% confluency by predigesting them in collagenase 2 (30 min) and trypsin (Thermo Fisher Scientific) digestion (20 min), and seeded at the same density. Medium change (DMEM + FBS) was performed every other day. At passage 3, 250,000 cells were transferred into 96 well v-bottom nonadherent plates (Greiner, Kremsmünster, Austria), centrifuged at 500 × g for 10 min to form pellets, and kept for 1 week in a chondropermissive culture medium without growth factors, comprising DMEM with 10% FBS, 60 µg/mL ascorbic acid phosphate (Sigma-Aldrich, St. Louis, USA), 40 µg/mL L-proline (Sigma-Aldrich), 1% nonessential amino acids (Gibco) and 1% penicillin/streptomycin (Sigma-Aldrich).

### 2.3 OA inflammation model and EV supplementation

In the inflammatory groups, TNFα (20 ng/mL; R & D Systems, Minneapolis, USA) was added with all medium changes to induce inflammation. The concentration was selected from previous studies for better comparison (24, 25). EV preparations (MSC-41.5-EV_i1_ and MSC-84-EV_i_) and hPL_4_-EVs as control were supplemented to the medium in the respective groups with a cell equivalent dose of 2 × 10^5^ (3.3 µl). This dose was effective in previous studies of other diseases (18, 26). Medium change including cytokine and EVs was performed five times. The medium was collected for further biochemical analysis.

### 2.4 Biochemical evaluation

Samples were prepared for biochemical analysis by proteinase K digestion (0.5 mg/mL; Roche, Basel, Switzerland) in phosphate buffer overnight. DNA was quantified using spectrofluorimetry with Hoechst dye solution 33528 (Applied Biosystems, Waltham, USA) against the standard calf thymus DNA (Sigma-Aldrich) (27). The pellets and medium were analyzed for glycosaminoglycan (GAG) content using a 1,9-dimethyl-methylene blue (DMMB; Sigma-Aldrich) dye-binding assay against the standard bovine chondroitin sulfate (Sigma-Aldrich), as described previously (28). DMMB interacts with the highly negatively charged sulfated GAGs resulting in a colored product that can be measured photometrically and is directly proportional to the concentration of sulfated GAGs in the sample. The DMMB assay detects all sulfated GAGs, including chondroitin sulfates (CS), keratan sulfates (KS), and heparan sulfates (HS). Collagen analysis was performed by determining the hydroxyproline concentration after acid hydrolysis using spectrophotometry with p-dimethylaminobenzaldehyde (Fluka) and chloramine-T (Sigma-Aldrich) (29). The medium was analyzed at all separate time points for nitric oxide (NO) content using a Griess diazotization reaction assay against the nitrite standard (Promega, Walldorf, Germany). IL-6 concentration was evaluated in the medium samples using a bovine IL-6 ELISA assay kit (Kingfisher Biotech, St. Paul, USA).

### 2.5 RNA extraction, reverse transcription, and gene expression analysis

Pellet samples from three different donors were pooled (n=4) and homogenized using a tissue-lyzer system (Qiagen, Hilden, Germany) in 1 mL TRI reagent (Molecular Research Center, Cincinnati, USA) for 10 min at 30 Hz. RNA was extracted using the precipitation method with bromochloropropane (BCP, Sigma-Aldrich) in a volume ratio of 1:10 for phase separation and RNA cleanup with a tissue-specific column-based extraction kit (Qiagen). Reverse transcription was performed with TaqMan^®^ reverse transcription reagents (Applied Biosciences) using 1 µg total RNA to generate cDNA. Gene expression was analyzed using a Real-Time PCR system (Applied Biosystems) with the TaqMan master mix and custom-made bovine primers and probes (Applied Biosystems) as previously described (30, 31). Bovine TaqMan assay was performed for the chondrogenic markers COL-II, aggrecan, proteoglycan 4 (PRG-4), cartilage oligomeric protein (COMP), dedifferentiation marker COL-I, hypertrophy marker COL-X, catabolic matrix metalloproteinases 3 (MMP-3), MMP-13, a disintegrin and metalloproteinase with thrombospondin motifs 4 (ADAMTS-4) and ADAMTS-5, vascular endothelial growth factor (VEGF) and the cytokine IL-8. Gene expression was measured relative to the endogenous control 18S ribosomal RNA. In a comparative analysis, the threshold cycle (CT) values were normalized to mean CT values of 18S (ΔCT) and normalized to day 0 (ΔΔCT). Relative mRNA expression was calculated using the 2^-ΔΔCT^ method.

### 2.6 Histology and immunohistochemistry

The samples were fixed in 70% methanol. For paraffin embedding, they were transferred to a carousel tissue processor for 24 h using the steps 70% ethanol, 96% ethanol, 100% ethanol, xylene, and paraffin. After paraffin embedding, the samples were cut into 5 µm sections using a microtome. Staining was performed using safranin O and Fast Green (Sigma-Aldrich) to evaluate cell morphology and extracellular matrix deposition. Slides were first stained with Weigert´s Iron Hematoxylin Stain Kit (Sigma-Aldrich) for 10 min, followed by 0.02% Fast Green (Sigma-Aldrich) in ultrapure (ddH_2_O) water for 6 min and 0.1% safranin O for 10 min. COL-I and COL-II deposition was evaluated using immunohistochemistry with the Vectastain ABC-based (avidin-biotin-complex; Vector Laboratories, Burlingame, USA) staining method as previously described, with antibodies against COL-I (Arcis, Warrington, United Kingdom) or COL-II (DSHB, Iowa, USA) and PBS as the negative control, followed by a biotinylated IgG antibody, DAB (3-3´-diaminobenzidine; Vector Laboratories) and Mayer hematoxylin (Sigma-Aldrich) as the counterstain (24). For all the immunostains, peroxidase activity was visualized using diaminobenzidine (ImmPACT DAB, Vector Laboratories). The pellets were semiquantitatively evaluated for COL-I and COL-II content in a pixel analysis against background stain. The percentage of stain inside the pellets was compared between the groups.

### 2.7 Statistical analysis

SPSS (v 24; IBM, Armonk, USA) was used for statistical analysis. The Kolmogorov-Smirnov test was used to test normal distribution. The nonparametric Wilcoxon-Mann-Whitney test was applied to test for significant differences among the groups as independent variables and analytical results as dependent groups. Significance was defined at p < 0.05. The graphical illustration was performed using GraphPad Prism 9 (GraphPad Software Inc., San Diego, USA).

## 3 Results

### 3.1 Experimental design

**Figure 1:**
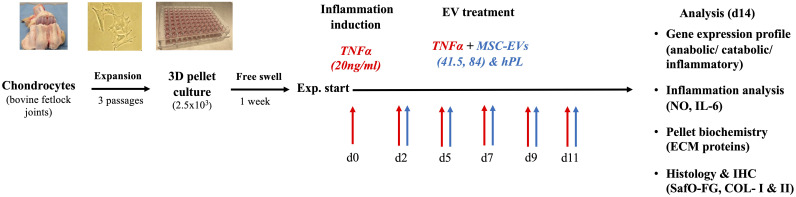
Schematic overview of the experimental design. The chondrocytes were isolated from bovine fetlock joints, expanded to passage 3, and transferred into a 3D pellet culture for chondrogenic differentiation. Cytokine supplementation with TNFα was performed within 14 days. MSC-EV treatment was started on day 2 with two different MSC-EV preparations (41.5-EV_i1_ and 84-EV_i_) and hPL_4_-EVs as a control. A broad analysis was performed on gene expression levels using RT-PCR and biochemical and histological analyses to evaluate the specific effect on inflammation and chondrocyte differentiation.

Chondrocytes were isolated from bovine fetlock joints, monolayer expanded to passage 3 (6–7 population doublings), and transferred into a 3D pellet culture. After 1 week of culture, the pellets were divided into eight different groups. Three different EV preparations (MSC-41.5-EV_i1_, MSC-84-EV_i_, and human platelet lysate (hPL_4_)) were supplemented and a non-EV control was selected. TNFα (20ng/ml) was added for simulation of the inflammatory surroundings. The medium was changed three times per week. The experiment was performed using three different donors (**Figure 1**) and terminated on day 14 for all groups.

### 3.2 MSC-EV preparations show different effects on anabolic and catabolic gene expression patterns

**Figure 2:**
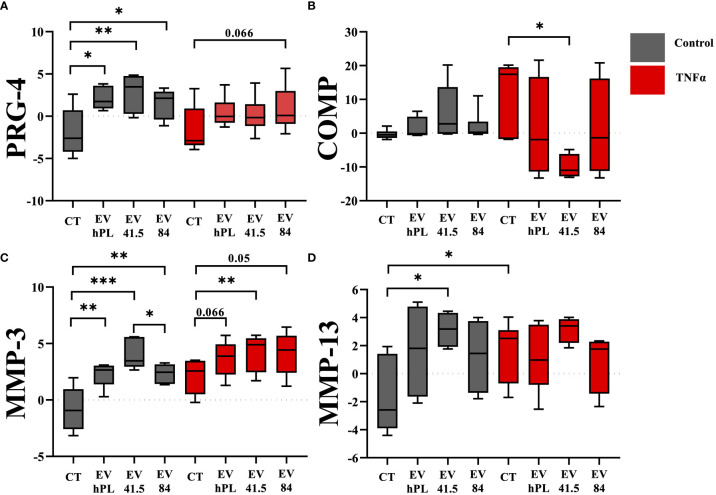
Effect of EV supplementation in a chondrocytes inflammation model with TNFα on mRNA levels of anabolic markers **(A)** proteoglycan 4 (PRG-4), **(B)** cartilage oligomeric matrix protein (COMP), and catabolic factors **(C)** matrix metalloproteinase 3 (MMP-3), and **(D)** MMP-13 relative to day 0. The results are transformed using natural logarithm and visualized in box plots. N=3 bovine donors; *p < 0.05, **p < 0.01, ***p < 0.001. con, control; EV, extracellular vesicle; hPL, human platelet lysate; EV 41.5 & EV 84, MSC-EV preparation 41.5-EV_i1_ & 84-EV_i_ from different donors; TNFα, tumor necrosis factor alpha.

A specific gene expression analysis with anabolic and catabolic key markers for the evaluation of the chondrogenic differentiation potential of articular chondrocytes was performed. Different patterns were detected between EV supplemented and control samples. MSC-EV and hPL-EV supplementation increased the expression of the anabolic marker PRG-4 compared to the untreated control (MSC-41.5-EV_i1_: p = 0.008; MSC-84-EV_i_: p = 0.029; hPL_4_-EV: p= 0.028; **Figure 2**). PRG-4 is a specific surface protein of cartilage and important for joint lubrication (32). However, this anabolic effect was not detected in inflammatory surroundings by supplementation of TNFα and only a trend for higher expression levels was detected by supplementation of MSC-84-EV_i_ (p = 0.066). Gene expression of cartilage oligomeric protein (COMP) as a marker for COL-II integrity was not different between EV supplementation and untreated control in the absence of TNFα. In contrast, combination of the supplementation of TNFα and MSC-41.5-EV_i1_ resulted in downregulation of COMP (p = 0.017). COL- II and aggrecan as important marker for chondrocyte differentiation did not show differences between the different treatment groups (**Supplementary Figure 2**). For the catabolic effects including the cleavage of extracellular matrix components gene expression analysis of the matrix metalloproteinases MMP-3 and MMP-13 were analyzed. Fourteen days of TNFα stimulation resulted in upregulation of the catabolic markers MMP-3 (p = 0.015) and MMP-13 (p = 0.026; **Figure 2**) compared to the untreated control. The supplementation of all MSC-EV preparations and hPL_4_-EVs increased MMP-3 expression in the presence and absence of TNFα. Interestingly, this catabolic pattern was increased to the highest by supplementation of MSC-41.5-EV_i1_ (p = 0.041). A similar gene expression pattern was also detected for the catabolic markers ADAMTS-4 and ADAMTS-5, whereas the hypertrophic marker VEGF was not stimulated by supplementation of TNFα (**Supplementary Figure 4**). Furthermore, MSC-41.5-EV_i1_ preparation increased MMP-13 expression (p = 0.019). No significant differences could be seen for dedifferentiation marker COL-I and hypertrophy marker COL-X (**Supplementary Figure 2**).

### 3.3 MSC-EVs increase total GAG production with higher retention and release into the medium

**Figure 3:**
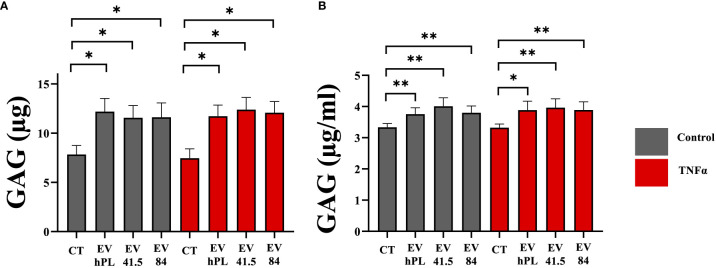
Quantitative analysis of **(A)** glycosaminoglycan (GAG) retention in a pellet culture and **(B)** GAG release into the medium of bovine passage 3 chondrocytes. The results are presented as the mean + SEM from three different donors (n =3). *p < 0.05, **p < 0.01. con, control; EV, extracellular vesicle; hPL, human platelet lysate; EV 41.5 & EV 84, MSC-EV preparation 41.5-EV_i1_ & 84-EV_i_ from different donors; TNFα, tumor necrosis factor alpha.

Glycosaminoglycans (GAG) are a main component of proteoglycans, which are important for the biomechanical characteristic of the extracellular matrix. The synthesis of glycosaminoglycans was significantly increased in all MSC-EV and hPL_4_-EV groups compared to control in presence and absence of TNFα supplementation (**Figure 3**). Both GAG retention as a marker for chondrogenic differentiation and GAG release in the medium were significantly higher than those of the control group. TNFα did not show any effect on GAG synthesis (**Figure 3**).

### 3.4 MSC-41.5-EV_i1_ increases inflammation, whereas MSC-84-EV_i_ exert anti-inflammatory effects

**Figure 4:**
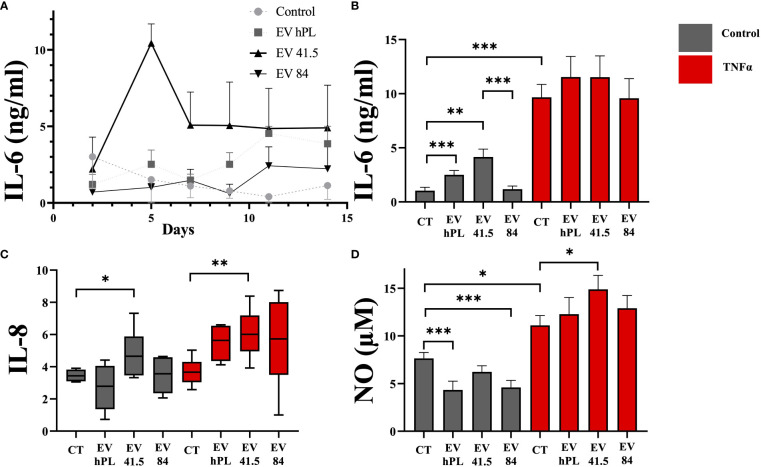
Effect of EV supplementation in a chondrocyte inflammation model with TNFα on inflammation markers. **(A)** IL-6 protein concentration in cell culture medium as kinetic evaluation without TNFα supplementation, **(B)** IL-6 absolute amount, **(C)** mRNA gene expression profile of IL-8 (relative to day 0), and **(D)** cumulative nitric oxide (NO) release into the medium. The results are presented as the mean + SEM from three different donors (n =3). *p < 0.05, **p < 0.01, ***p < 0.001. con, control; EV, extracellular vesicle; hPL, human platelet lysate; EV 41.5 & EV 84, MSC-EV preparation 41.5-EV_i1_ & 84-EV_i_ from different donors; TNFα, tumor necrosis factor alpha.

Inflammation is an important factor in disease progression of osteoarthritis, which is mediated by the proinflammatory cytokines such as TNFα (25). A broad inflammation marker panel was selected to analyze the effect of MSC-EV supplementation in inflammatory (including 20ng/ml TNFα) and non-inflammatory surroundings. TNFα treatment increased IL-6 and NO release into the medium. Gene expression of IL-8 was not upregulated by TNFα compared to control. Supplementation of MSC-41.5-EV_i1_ increased the release of the pro-inflammatory cytokine IL-6 with peak values on day 5 (**Figure 4A**) in the absence of TNFα stimulation. Total IL-6 production by MSC-41.5-EV_i1_ and hPL_4_-EVs was significantly higher than that of the control (control: 1.0 ± 0.3; MSC-41.5-EV_i1_:4.2 ± 0.7; hPL_4_-EV: 2.5 ± 0.4; [ng/ml]; p < 0.001), in contrast to MSC-84-EV_i_ which was comparable to the control. TNFα strongly induced IL-6 production by chondrocytes (control: 1.0 ± 0.3; control TNFα: 9.7 ± 1.2; [ng/ml] p < 0.001) without any differences between the different treatments (**Figure 4B**). In addition, MSC-41.5-EV_i1_ supplementation upregulated chondrocyte mRNA gene expression of IL-8 in the control and TNFα groups (control: p = 0.022; control TNFα: p = 0.005; **Figure 4C**). The inflammation marker nitric oxide (NO) was significantly lower than the control by treatment with MSC-84-EV_i_ and hPL_4_-EV, but not in the MSC-41.5-EV_i1_ group (**Figure 4D**). TNFα supplementation induced NO release into the medium (control: 7.6 ± 0.6; control TNFα: 11.1 ± 1; [µM] p = 0.013). Further, MSC-41.5-EV_i1_ induced NO production in the presence of TNFα (MSC-41.5-EV_i1:_ 14.9 ± 1.5; [µM] p = 0.013).

### 3.5 Chondrocyte matrix synthesis and differentiation are increased in MSC-84-EV_i_ and all MSC-EV-treated inflammatory surroundings

**Figure 5:**
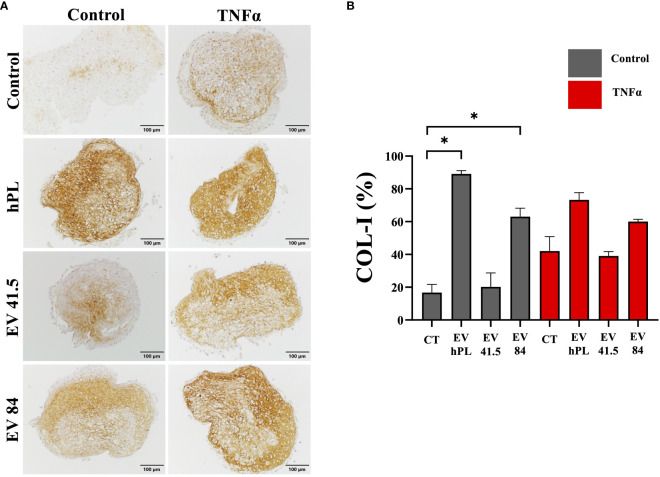
**(A)** Immunohistochemistry of COL-I and **(B)** semi-quantitative analysis of stain intensity compared to background stain (hematoxylin and eosin) in a pellet culture of bovine passage 3 chondrocytes. **(A)** Scale bar = 100 µm. **(B)** The results are presented as the mean + SEM from four pellets of one representative donor (n =1). *p < 0.05. con, control; EV, extracellular vesicle; hPL, human platelet lysate; EV 41.5 & EV 84, MSC-EV preparation 41.5-EV_i1_ & 84-EV_i_ from different donors; TNFα, tumor necrosis factor alpha.

**Figure 6:**
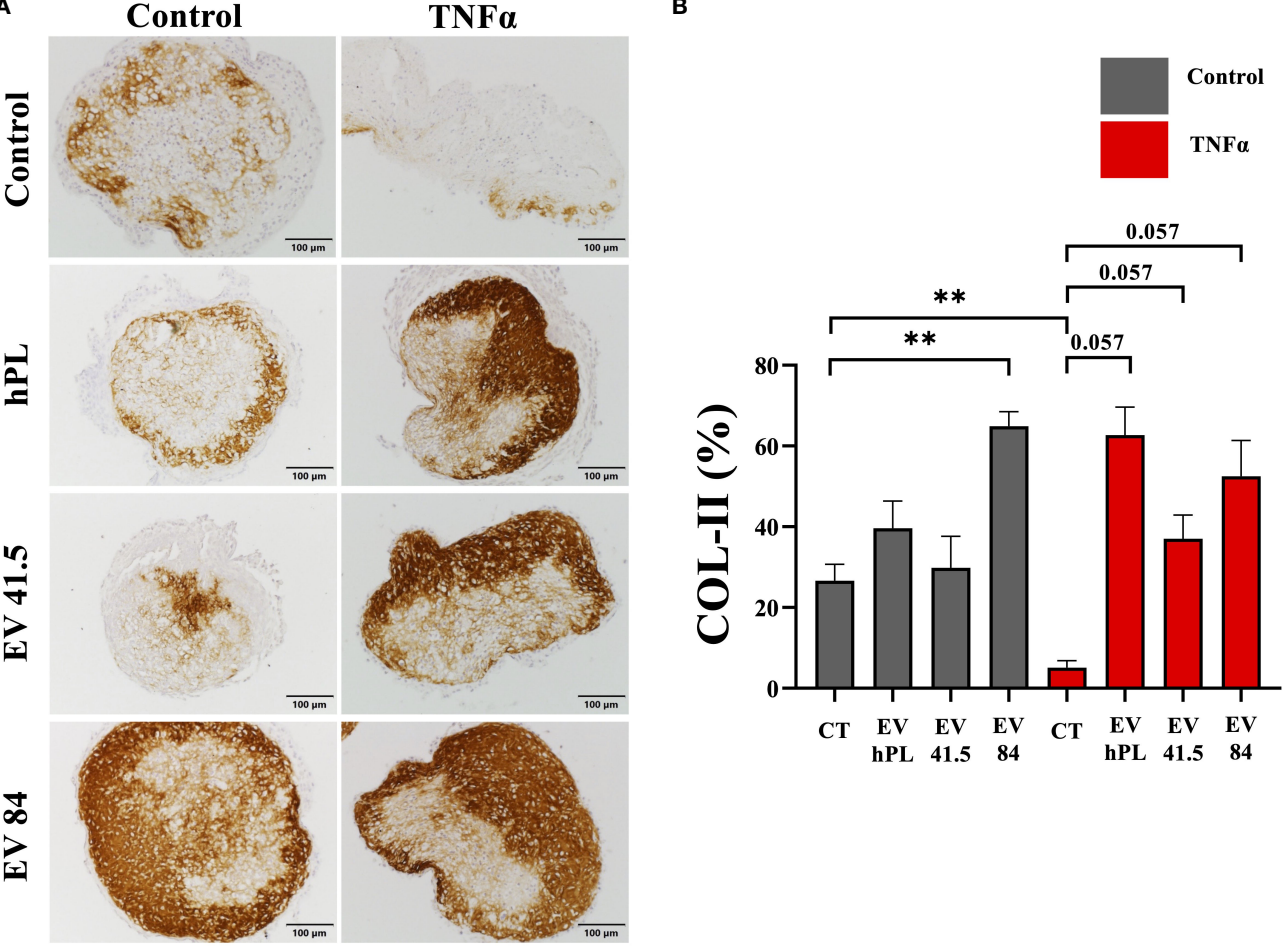
**(A)** Immunohistochemistry of COL-II and **(B)** semi-quantitative analysis of stain intensity compared to background stain (hematoxylin and eosin) in a pellet culture of bovine passage 3 chondrocytes. **(A)** Scale bar = 100 µm. **(B)** The results are presented as the mean + SEM from four pellets of one representative donor (n = 1). **p < 0.01. con, control; EV, extracellular vesicle; hPL, human platelet lysate; EV 41.5 & EV 84, MSC-EV preparation 41.5-EV_i1_ & 84-EV_i_ from different donors; TNFα, tumor necrosis factor alpha.

The result of matrix production was analyzed by histology and immunohistochemistry to evaluate chondrogenic differentiation effects and integrity of the resulting tissue. As a general overview stain safranin O/Fast Green was selected evaluating extracellular matrix integrity and quality. P3 chondrocyte pellet constructs showed a matrix with a low red stain in the safranin O/Fast Green staining indicating a reduced level of proteoglycans compared with the native tissue and a strong green stain of collagen (**Supplementary Figure 3A**). All pellets were the same size in diameter regardless of treatment (**Supplementary Figure 3B**). An immunohistochemical analysis of COL-I and COL-II was performed for evaluation of the re-differentiation status. In physiological conditions predominantly COL-II is evident in hyaline cartilage, whereas COL-I indicates chondrocyte dedifferentiation. In this experiment the extracellular matrix of untreated control samples showed a weak staining for COL-I and stronger staining for COL-II as a sign of chondrogenic differentiation (**Figures 5** **,** **6**). TNFα supplementation increased COL-I synthesis in the control group, whereas COL-II was significantly lowered (p = 0.029). The treatment with MSC-EV preparations resulted in a different collagen distribution in the pellet constructs. MSC-84-EV_i_ significantly increased COL-I and COL-II retention in the absence of TNFα. Interestingly, the combination of TNFα and MSC-EV preparations (41.5-EV_i1_, 84-EV_i_, and hPL_4_; **Figure 6**) elevated COL-II retention. However, this effect was only a trend in semiquantiative analysis (p = 0.057).

## 4 Discussion

### 4.1 Key findings

**Figure 7:**
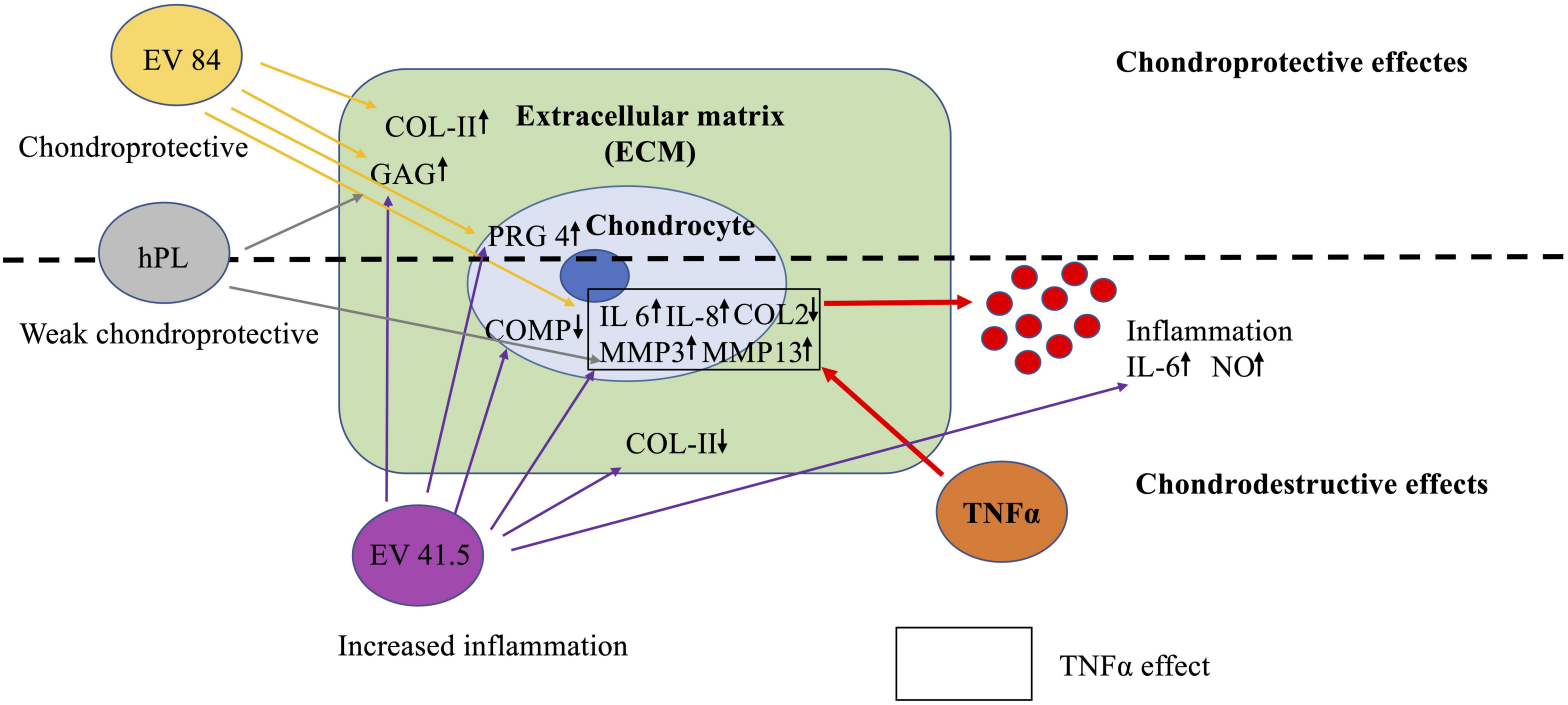
Key findings on the effects of MSC-EV supplementation in a chondrocyte inflammation model with TNFα. MSC-EV preparations differ in chondrogenic potential. EV 41.5 (41.5-EV_i1_) increases inflammation, while EV 84 (84-EV_i_) has a more anabolic potential. The control EVs from human platelet lysate (hPL, hPL-EV) have a minor effect on chondrogenesis.

This pilot study focused on the effect of MSC-EV preparations from different healthy donors on cartilage regeneration in an inflammation model with the pro-inflammatory cytokine TNFα. Interestingly, there was a strong difference in quantitative and qualitative analysis between the 3D chondrocyte pellet constructs supplemented with different MSC-EV preparations (**Figure 7**). MSC-84-EV_i_ treatment increased chondrogenic differentiation, while MSC-41.5-EV_i1_ stimulated inflammation. EVs from hPL as negative control had a minor effect on cartilage regeneration.

### 4.2 Therapeutic potential in chondrocyte inflammation models

This study analyzed for the first time a therapeutic approach in chondrocyte pellet culture (physiological 3D surroundings) applying multiple doses of MSC-EV preparations within 12 days of culture. Chondrocyte inflammation models are well established and often used to analyze therapeutic potential. TNFα and IL-1ß are major pro-inflammatory cytokines in inflammatory joint diseases, including rheumatoid arthritis and osteoarthritis, and are discussed as potential diagnostic markers for disease progression (2, 33). Pro-inflammatory cytokines have a catabolic effect on chondrocytes (34). Moreover, other cells of the knee joint can have a negative effect on cartilage regeneration in OA via EV cargo. Kato et al. demonstrated that EVs from IL-1ß-stimulated synovial fibroblasts highly increased MMP-13 expression (35). A coculture of M1 macrophages and OA chondrocytes induced inflammation with increased levels of TNFα and IL-1ß (36). This effect could be lowered by EVs from platelet-rich plasma. MSC-EV effects on chondrocytes in inflammation models were previously described. Interestingly all studies showed a therapeutic effect on modulating inflammation and increasing chondrogenesis. IL-1ß was used most frequently to induce a pro-inflammatory condition *in vitro* (13, 37–42). Nevertheless, TNFα plays a major role in OA progression, and was also assessed in several studies (43–45). However, previous studies did not investigate the MSC-EV effect in physiological 3D surroundings, but in monolayer culture for a short period (< 72 h). Another advantage of the present model is the pretreatment with the pro-inflammatory cytokine TNFα, while other studies started cytokine application and treatment at the same time.

Vonk et al. (43) investigated the MSC-EV effect of human BM-MSC with a similar cell equivalent dose (two donors) on human passaged chondrocytes in monolayer culture for 48 h in an inflammation model with TNFα (10 ng/mL). MSC-EV supplementation resulted in the inhibition of the NF-κB pathway resulting in the downregulation of COX-2 and the gene expression of pro-inflammatory cytokines IL-1α, IL-1ß, IL-6, IL-8, and IL-17. After a single MSC-EV treatment, they cultivated chondrocytes in fibrin for 4 weeks and detected a higher proteoglycan deposition using safranin O/Fast Green stain and an increased GAG/DNA ratio in all MSC-EV groups. Both MSC-EV preparations showed a similar regenerative anti-inflammatory potential in the chondrocyte culture. The effect on matrix deposition is consistent with our findings. Nevertheless, we demonstrated differences in the functional phenotype of different MSC-EV preparations.

Hotham et al. (44) analyzed the therapeutic potential of targeting inflammation by equine BM-MSCs in an *in vitro* inflammation model with TNFα and IL-1ß. They demonstrated an anticatabolic effect of equine BM-MSCs with significant reduction compared to control on the gene expression level of MMP-13 and ADAMTS-4 in monolayer expanded equine chondrocytes. However, there was no correlation with protein level. In our study, MSC-41.5-EV_i1_ stimulated chondrocyte MMP-13 expression, while 84-EV_i_ did not show any catabolic effect in the presence and absence of TNFα. Another equine model was used by Arevalo-Turrubiarte et al. (45), who also focused on both TNFα and IL-1ß. They analyzed MSC-EVs from different origins (bone marrow, synovium, and fat). They reported only a reduction in the levels of TIMP-1 and TIMP-3 in the presence of TNFα and a reduced expression of MMP-13 in the IL-1ß model for BMMSC-EVs. The overall effects were weak.

### 4.3 Effect of different MSC-EV preparations

In contrast to the previously published studies, we detected significant differences in the therapeutic potential of different MSC-EV donor preparations with increased inflammation by MSC-41.5-EV_i1_ and a chondrogenic stimulus by MSC-84-EV_i_. It can be claimed that the current evidence is a result of a certain systematic bias in publishing positive data and not publishing adverse effects. This heterogenous effect may contribute to the fact that the MSC-based therapeutic disease-modifying potential of preclinical *in vitro* and *in vivo* studies could not be demonstrated in clinical trials so far (9). In addition, MSC-EV preparations of this study did not show differences in transcriptome and proteome analyses. We have previously reported that the therapeutic potential of MSC-EVs can differ between donors without differences in MSC and EV characteristic analysis on proteomic and transcriptomic level (26, 46). There, we performed a broad miRNA and proteome analysis in another setting of an acute graft-versus-host-disease (aGVHD) model, where different MSC-EV preparations were applied, showing altered therapeutic activity in a mouse model (47). For functional characterization of MSC-EVs, an innovative approach is a mixed lymphocyte reaction assay (18, 48). Mononuclear cells from 12 different donors were used to produce an allogeneic immune response. MSC-EV preparations were used to modulate this effect. Immunomodulatory active preparations were selected and used to demonstrate their superior effect to suppress GvHD in an *in vivo* mouse model (48). Although observing differences between the hPL-EVs and MSC-EVs, no biomarker could be identified to discriminate therapeutically active from non-active EV preparations. As the model of OA deals with another potential mode of action, a suitable correlation of given potency/functional assays needs to be defined. To the best of our knowledge, there is no functional assay available to define the regenerative therapeutic potential in cartilage inflammation. This chondrocyte inflammation model could be used to characterize EVs and select the preparations with the highest therapeutic potential. Therefore, despite its pilot character, we want to share our new data to make the EV community aware of the potential of our *in vitro* system to study the chondrogenic effect of MSC-EVs. We believe that this topic can only be addressed in an interdisciplinary and collaborative manner due to the mentioned heterogeneity.

Moreover, EV cargo has been analyzed widely. Transcriptome and proteome analysis is of specific importance. There is a current debate on whether protein cargo or RNA would enable the therapeutic efficacy of MSC-EV (49). On average, the range of encapsulated RNA is between 200 and 400 nts, which is too short for protein-coding information (49). Nevertheless, micro RNA (miRNA) is claimed to play an important role as a mediator in the mechanism of chondrogenic stimulation (50–52). For instance, Hu et al. (53) reported that miR-355-3p which regulates P21-activated kinase and promotes TGF-β signaling, was overexpressed in the late stages of OA by human adipose-derived stem cells and chondrocytes. Wang et al. (54) reported a similar finding for miR-135b, stimulating TGF-ß1 expression. Furthermore, miRNA in cartilage, such as miR-9, miR-38, and miR-146, which are potential markers for osteoarthritis, were identified to increase inflammatory cartilage degradation (55).

Regarding protein level, Thomas et al. demonstrated increased cartilage repair through wnt3A using exosomes as a delivery vehicle (56). Nevertheless, in our study, we could not identify differences in protein and miRNA levels (data not shown).

A lack of standardization also affects current evidence on the therapeutic potential of MSC-EVs. The international society of extracellular vesicles introduced the MISEV criteria in 2018 for better characterization of specific EV preparations, which included the demand for reporting size and concentration (NTA), EV-specific proteins (western blotting), and EV morphology (electron microscopy) (22). Nevertheless, a large proportion of all preclinical studies dealing with MSC-EVs do not fulfill the criteria (17). Only 45% of the studies dealing with the MSC effect in chondrocyte inflammation models were in line with the MISEV 2018 criteria (see papers mentioned above). However, there is a lack of systematic reporting of the EV dose. In our study, we used the cell equivalent dose according to MSC-EV production over time. Many studies also report the particle amount (NTA), which makes a dose comparison difficult. NTA counts the number of nanoparticles, but cannot differentiate between EV and nonEV particles. Therefore, it is not a clear dose of the specific EVs.

Heterogeneity of MSC cell source and isolation technique can have an effect on the functional phenotype of MSCs, which can directly affect the therapeutic potential of secreted EVs (57). However, specific musculoskeletal diseases of the MSC donor, such as osteoarthritis and osteoporosis can also have an effect on the therapeutic potential of MSC-EVs. This is important for further implementation in clinics.

### 4.4 Limitations and outlook

This pilot study used a bovine chondrocyte pellet model to evaluate the therapeutic potential of human MSC-EVs. Bovine chondrocytes have the advantage to be more standardized than human OA chondrocytes in the analysis of the effect of TNFα. There is no evidence suggesting that chondrocytes show significant immunoactivity. Nevertheless, xenogenic effects cannot be fully excluded. In our study, we used passaged chondrocytes (P3) that were dedifferentiated through cultivation, which was evident by a loss of proteoglycans, decreased COL-II deposition, and increased synthesis of COL-I compared to non-passaged chondrocytes (P0). The chondrogenic differentiation was enhanced by all EV preparations. An application of EV preparations in cartilage repair, e.g., autologous chondrocyte implantation could be an innovative approach to further stimulate chondrocyte differentiation. In this context, it would be of great interest to gain more knowledge about the molecular effects of EVs on the molecular composition, tissue integrity, and function of chondrocyte pellets. In addition, a major challenge of current EV research is the high heterogeneity of EV preparations and their differences in functional phenotype without markers on transcriptomic and proteomic level, that are able to select chondroprotective EV preparations. Therefore, standardization of the EV preparation evaluation and development of functional tests to detect EV preparations with chondrogenic effects are essential. As next step, *in vivo* studies of EV potency evaluation in osteoarthritic surroundings are necessary for further translation into clinics.

### 4.5 Conclusion

This pilot study focused on the therapeutic potential of MSC-EVs in a 3D chondrocyte inflammation model. MSC-EVs supplementation demonstrated a positive chondrogenic stimulation potential, but were different in functional phenotype depending on the donor. MSC-84-EV_i_ treatment showed a high chondrogenic potential, whereas MSC-41.5-EV_i1_ stimulated inflammation and degradation. There is an urgent need to characterize and develop new functional markers indicating MSC-EVs with high therapeutic potential in chondrocyte inflammation.

## Data availability statement

The raw data supporting the conclusions of this article will be made available by the authors, without undue reservation.

## Author contributions

All authors listed made a substantial, direct, and intellectual contribution to the work, and approved it for publication.

## Supplementary material

Supplementary Figure 1 Characterization of MSC-EVs by ImageStreamX flow cytometry. EVs from hPL, 41.5-EV_i1_, and 84-EV_i_ were analyzed using imaging flow cytometry for CD9, CD59, CD63 and CD81. **(A)** From all recorded signals (1st plot from left), signals not showing spot counts or signal multiplets were excluded (2nd plot from left). In the four representative plots on the right, side scatter (SSC) intensities of single objects are plotted against the fluorescence intensities of CD9^+^ (labeled with PE), CD59^+^ (labeled with FITC), CD63^+^ (labeled with APC) or CD81^+^ (labeled with FITC) objects. **(B)** Results of the preparations are given in objects/mL for CD9^+^, CD59^+^, CD63^+^, and CD81^+^. The results are presented as the mean + SD.

Supplementary Figure 2 Influence of EV supplementation in an inflammation model with TNFα on mRNA levels of dedifferentiation marker **(A)** COL-I, hypertrophy marker **(B)** COL-X, cartilage differentiation factor **(C)** COL-II and **(C)** aggrecan (ACAN) relative to day 0. Results are transformed by natural logarithm and visualized in box plots. *p < 0.05, **p < 0.01, ***p < 0.001. con, control; EV, extracellular vesicle; hPL, human platelet lysate; EV 41.5 & EV 84, MSC-EV preparation 41.5-EV_i1_ & 84-EV_i_ from different donors; TNFα, tumor necrosis factor alpha.

Supplementary Figure 3
**(A)** Histology (safranin O/Fast Green) and **(B)** pellet diameter (µm) of bovine passage 3 chondrocytes. **(A)** Scale bar 100µm. **(B)** Results are the mean + SEM from 3 different donors. *P < 0.05, **P < 0.01, ***P < 0.001. con, control; EV, extracellular vesicle; hPL, human platelet lysate; EV 41.5 & EV 84, MSC-EV preparation 41.5-EV_i1_ & 84-EV_i_ from different donors; TNFα, tumor necrosis factor alpha. Safranin O Fast Green and Pellet diameter.

Supplementary Figure 4 Influence of EV supplementation in an inflammation model with TNFα on mRNA levels of catabolic marker **(A)** a disintegrin and metalloproteinase with thrombospondin motifs 4 (ADAMTS-4), **(B)** ADAMTS-5 and hypertrophy marker **(C)** vascular endothelial growth factor (VEGF) relative to day 0. Results are transformed by natural logarithm and visualized in box plots. *p < 0.05, **p < 0.01, ***p < 0.001. con, control; EV, extracellular vesicle; hPL, human platelet lysate; EV 41.5 & EV 84, MSC-EV preparation 41.5-EV_i1_ & 84-EV_i_ from different donors; TNFα, tumor necrosis factor alpha.
