# Supplementary material for: Immunomodulatory potential of mesenchymal stromal cell-derived extracellular vesicles in chondrocyte inflammation
Source: Front Immunol. 2023 Jul 26;14:1198198. doi: 10.3389/fimmu.2023.1198198 (PMC10410457; doi:10.3389/fimmu.2023.1198198)
Supplement: Supplementary file 1 [file DataSheet_1.docx]

# Supplementary Methods

**Imaging flow cytometry for MSC-EV characterization**

Unless mentioned otherwise, the staining of the EV preparations was performed as described previously (1,2). Briefly, 5 µL of PEG-prepared EV samples were labelled with 0.5 µL PE-conjugated mouse anti-human CD9 (EXBIO), FITC-conjugated mouse anti-human CD59 (BD Biosciences), APC-conjugated mouse anti-human CD63 (EXBIO) or FITC-conjugated mouse anti-human CD81 (Beckman Coulter). Unstained samples and buffer controls without EVs but with antibodies were used as controls. All samples were incubated 1 h in the dark at RT and diluted 100-fold for anti-CD9 and 40-fold for anti-CD59, anti-CD63 or CD81 with PBS (pH 7.4; Gibco) before analysis. Next, without further washing, samples were analyzed with an ImageStreamX Mark II instrument (Amnis/Luminex, Seattle). All data were acquired with 5 min acquisition time at 60x magnification at low flow rate (0.3795 ± 0.0003 μL/min) and with removed beads option deactivated as described previously. Data analysis was performed using IDEAS software version 6.2 as previously described (2). All fluorescent events were plotted against the side scatter. Images were analyzed for coincidences by using the spot counting feature. Events with multiple spots were excluded from further analysis.

References:

1. Tertel, T., Bremer, M., Maire, C., Lamszus, K., Peine, S., Jawad, R., Andaloussi, S.E.L., Giebel, B., Ricklefs, F.L., and Görgens, A. (2020a). High-Resolution Imaging Flow Cytometry Reveals Impact of Incubation Temperature on Labeling of Extracellular Vesicles with Antibodies. Cytometry Part A *97*, 602-609.
2. Tertel, T., Gorgens, A., and Giebel, B. (2020b). Analysis of individual extracellular vesicles by imaging flow cytometry. Methods Enzymol *645*, 55-78.
