# Supplementary figures and images for: Immunomodulatory potential of mesenchymal stromal cell-derived extracellular vesicles in chondrocyte inflammation
Source: Front Immunol. 2023 Jul 26;14:1198198. doi: 10.3389/fimmu.2023.1198198 (PMC10410457; doi:10.3389/fimmu.2023.1198198)

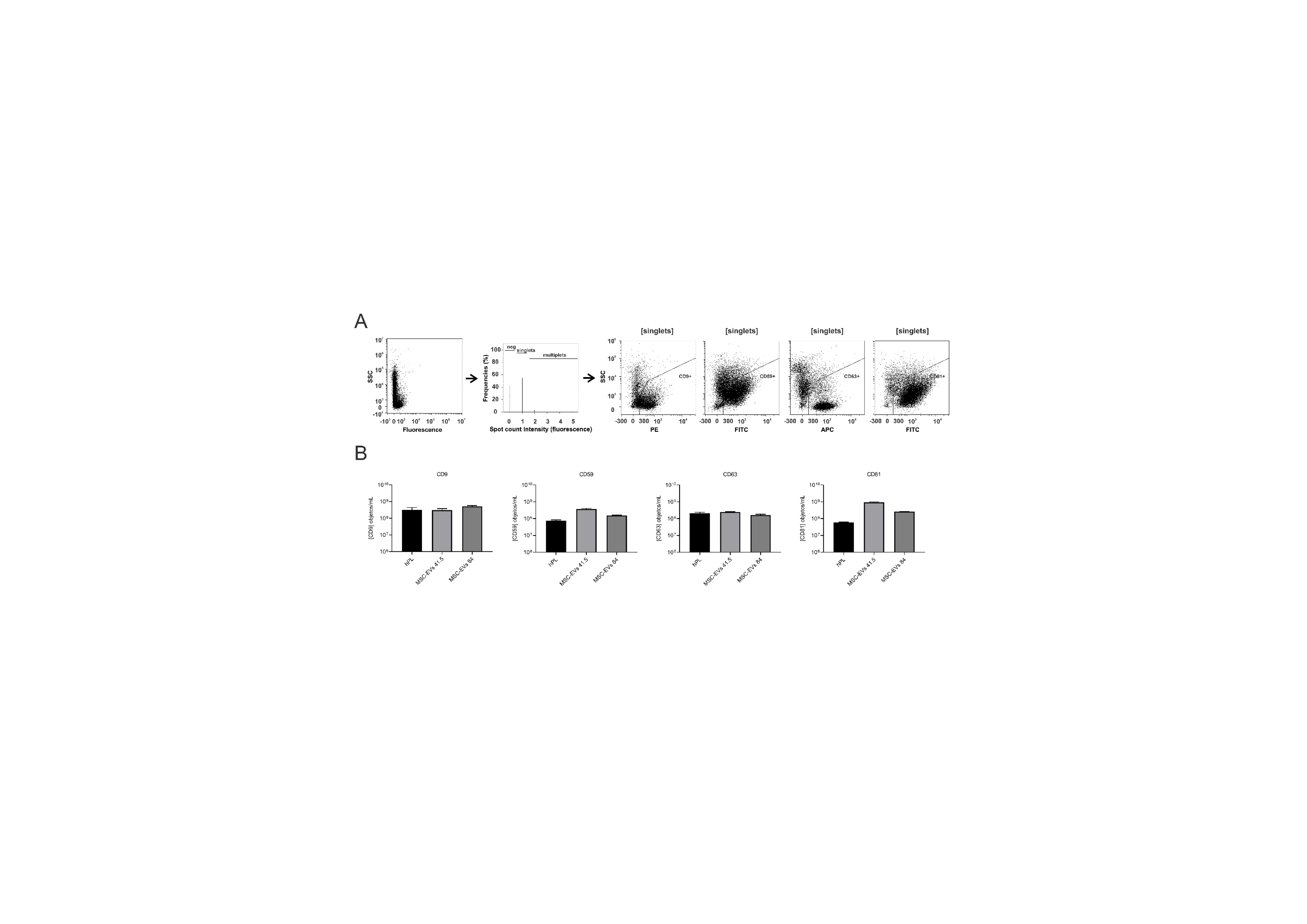

Supplement: Supplementary Figure 1 — Characterization of MSC-EVs by ImageStreamX flow cytometry. EVs from hPL, 41.5-EVi1, and 84-EVi were analyzed using imaging flow cytometry for CD9, CD59, CD63 and CD81. (A) From all recorded signals (1st plot from left), signals not showing spot counts or signal multiplets were excluded (2nd plot from left). In the four representative plots on the right, side scatter (SSC) intensities of single objects are plotted against the fluorescence intensities of CD9+ (labeled with PE), CD59+ (labeled with FITC), CD63+ (labeled with APC) or CD81+ (labeled with FITC) objects. (B) Results of the preparations are given in objects/mL for CD9+, CD59+, CD63+, and CD81+. The results are presented as the mean + SD. [file Image_1.jpg]

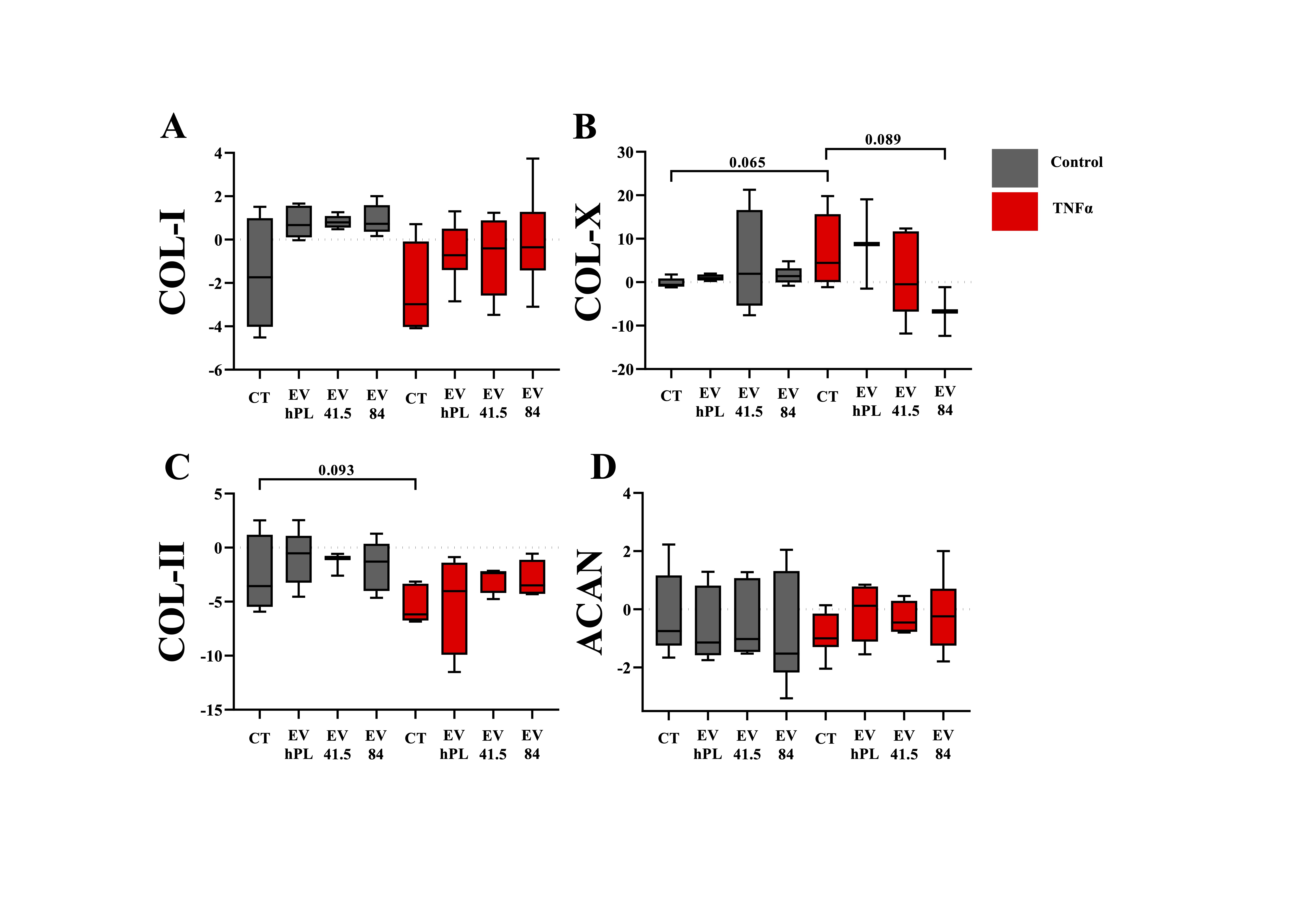

Supplement: Supplementary Figure 2 — Influence of EV supplementation in an inflammation model with TNFα on mRNA levels of dedifferentiation marker (A) COL-I, hypertrophy marker (B) COL-X, cartilage differentiation factor (C) COL-II and (C) aggrecan (ACAN) relative to day 0. Results are transformed by natural logarithm and visualized in box plots. *p < 0.05, **p < 0.01, ***p < 0.001. con, control; EV, extracellular vesicle; hPL, human platelet lysate; EV 41.5 & EV 84, MSC-EV preparation 41.5-EVi1 & 84-EVi from different donors; TNFα, tumor necrosis factor alpha. [file Image_2.jpg]

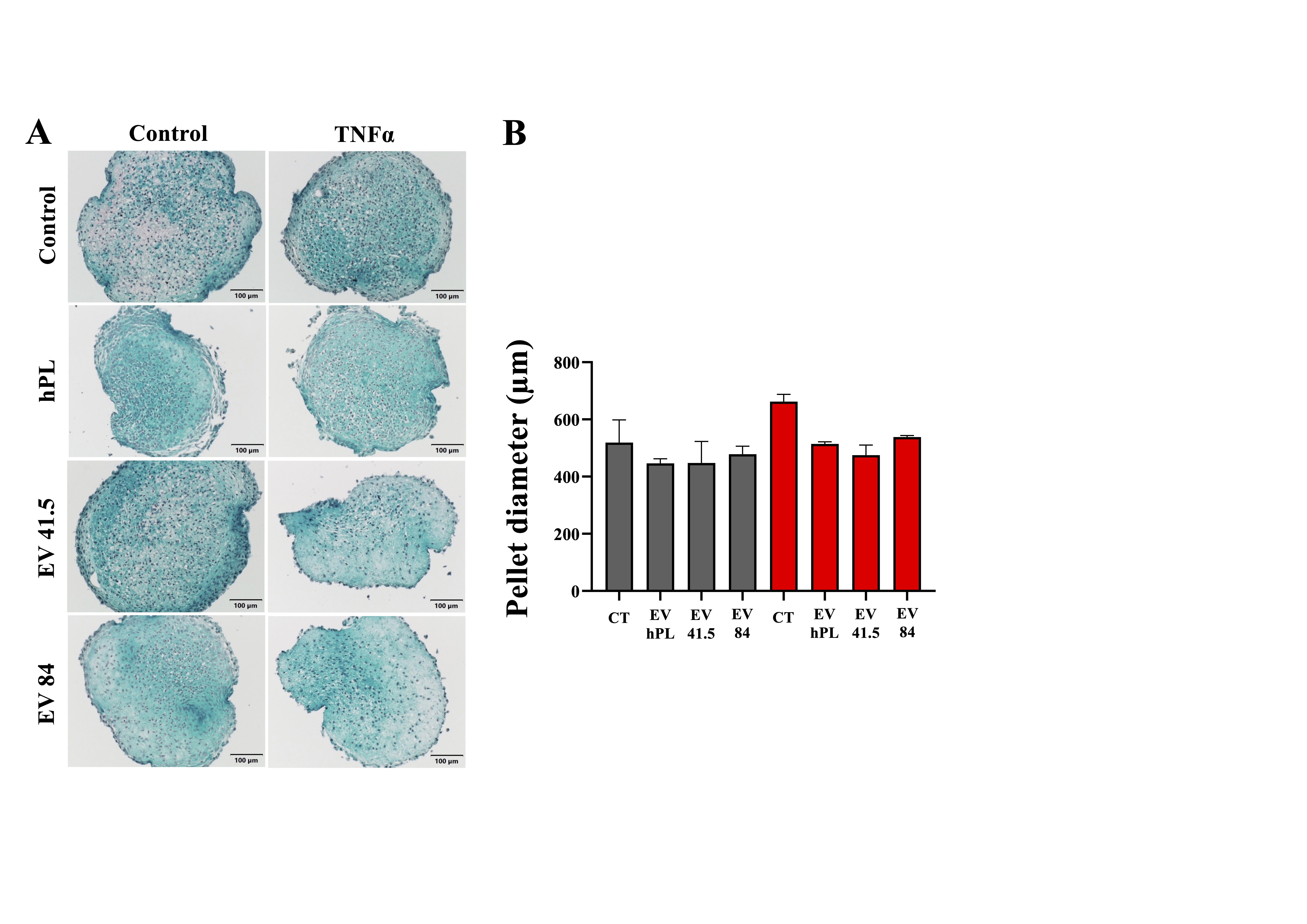

Supplement: Supplementary Figure 3 — (A) Histology (safranin O/Fast Green) and (B) pellet diameter (µm) of bovine passage 3 chondrocytes. (A) Scale bar 100µm. (B) Results are the mean + SEM from 3 different donors. *P < 0.05, **P < 0.01, ***P < 0.001. con, control; EV, extracellular vesicle; hPL, human platelet lysate; EV 41.5 & EV 84, MSC-EV preparation 41.5-EVi1 & 84-EVi from different donors; TNFα, tumor necrosis factor alpha. Safranin O Fast Green and Pellet diameter. [file Image_3.jpg]

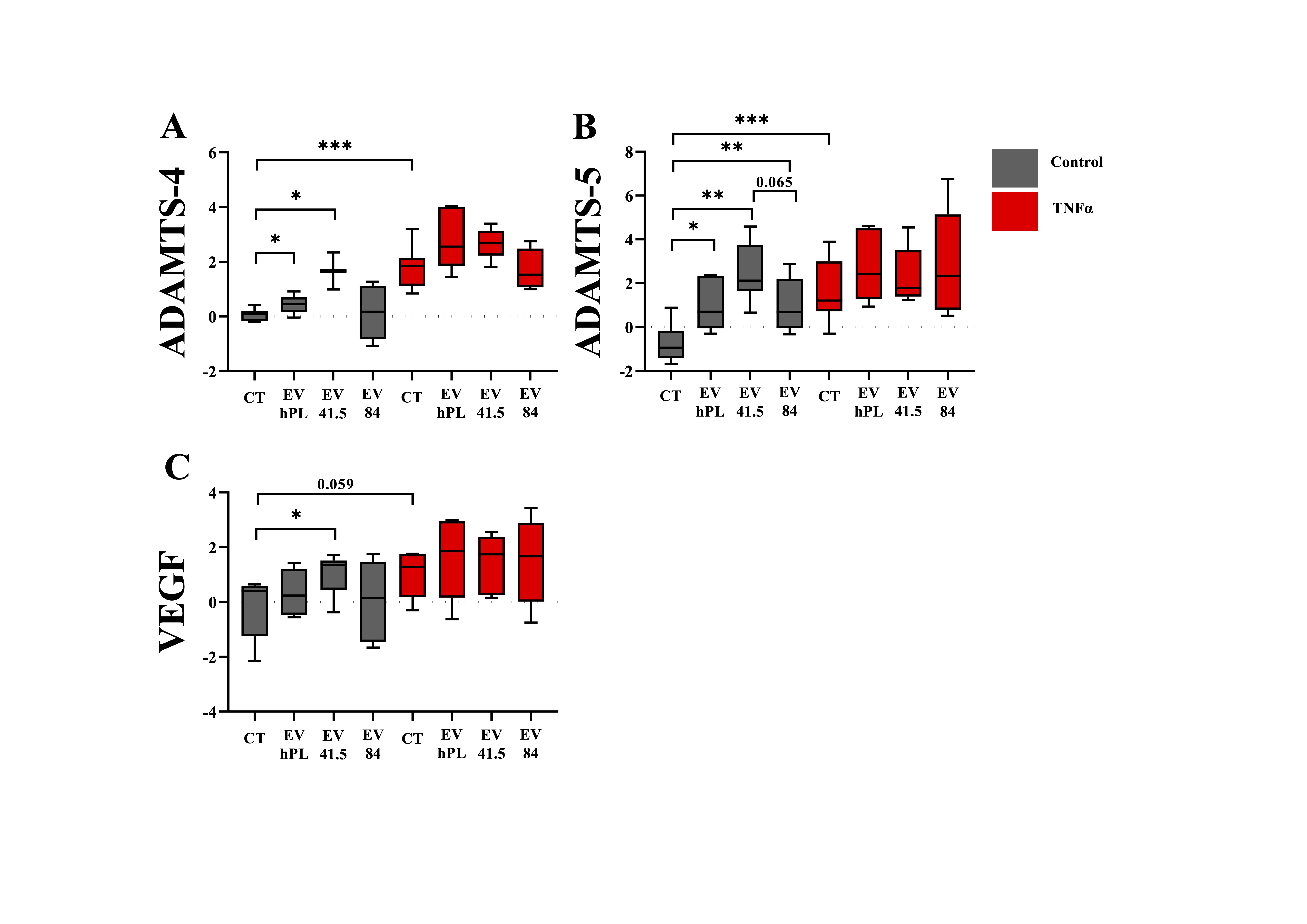

Supplement: Supplementary Figure 4 — Influence of EV supplementation in an inflammation model with TNFα on mRNA levels of catabolic marker (A) a disintegrin and metalloproteinase with thrombospondin motifs 4 (ADAMTS-4), (B) ADAMTS-5 and hypertrophy marker (C) vascular endothelial growth factor (VEGF) relative to day 0. Results are transformed by natural logarithm and visualized in box plots. *p < 0.05, **p < 0.01, ***p < 0.001. con, control; EV, extracellular vesicle; hPL, human platelet lysate; EV 41.5 & EV 84, MSC-EV preparation 41.5-EVi1 & 84-EVi from different donors; TNFα, tumor necrosis factor alpha. [file Image_4.jpg]
